# Cancer care in Brazil: structure and geographical distribution

**DOI:** 10.1186/s12885-019-6190-3

**Published:** 2019-10-23

**Authors:** Mario Jorge Sobreira da Silva, Gisele O’Dwyer, Claudia Garcia Serpa Osorio-de-Castro

**Affiliations:** 1 National Cancer Institute, Rua Marquês de Pombal, 125 – 3° andar – Centro, Rio de Janeiro, RJ Zip code: 20230-240 Brazil; 2 Sergio Arouca National School of Public Health, Oswaldo Cruz Foundation, Rio de Janeiro, Brazil

**Keywords:** Cancer, Health policies, Health services

## Abstract

**Background:**

The organisation and systematisation of health actions and services are essential to ensure patient safety and the effectiveness and efficiency of cancer care. The objective of this study was to analyse the structure of cancer care envisaged in Brazilian norms, describe the types of accreditations of cancer services and their geographic distribution, and determine the planning and evaluation parameters used to qualify the health units that provide cancer care in Brazil.

**Methods:**

This observational study identified the current organisation of cancer care and other health services that are accredited by Brazil’s national health system (SUS) for cancer treatment as of February 2017. The following information was collected from the current norms and the National Registry of Health Establishments: geographic location, type of accreditation, type of care, and hospital classification according to annual data of the number of cancer surgeries. The adequacy of the number of licensed units relative to population size was assessed. The analysis considered the facilitative or restrictive nature of policies based on the available rules and resources.

**Results:**

The analysis of the norms indicated that these documents serve as structuring rules and resources for developing and implementing cancer care policies in Brazil. A total of 299 high-complexity oncology services were identified in facilities located in 173 (3.1%) municipalities. In some states, there were no authorised services in radiotherapy, paediatric oncology and/or haematology-oncology. There was a significant deficit in accredited oncology services.

**Conclusions:**

The parameters that have been used to assess the need for accredited cancer services in Brazil are widely questioned because the best basis of calculation is the incidence of cancer or disease burden rather than population size. The results indicate that the availability of cancer services is insufficient and the organisation of the cancer care network needs to be improved in Brazil.

## Background

Cancer is a group of diseases of multifactorial origin with increasing worldwide incidence and mortality, thus necessitating intersectional actions for its control when there are limited financial resources [1]. Cancer treatment is multimodal, involving surgery, radiotherapy, and chemotherapy, and is expensive [2]. However, there are large gaps in cancer treatment outcomes because of differences among countries in socioeconomic development and access to health services [3]. A fundamental mechanism for ensuring patient safety and the effectiveness and efficiency of cancer care is the organisation and systematisation of health actions and services [4–6].

According to the International Agency for Research on Cancer (IARC), in 2018 the estimated global incidence of cancer was 18.1 million cases, and the estimated mortality from cancer was 9.6 million people [1]. In Brazil, it is estimated that approximately 600,000 new cases of cancer will occur annually in 2018 and 2019 [7].

From the beginning of the twentieth century to 1937, cancer care in Brazil was largely absorbed by philanthropic institutions. That year, the National Cancer Institute was founded, with a clear role in setting policy, but status quo of care remained the same. In 1980, with the founding of SUS, the cancer care network was drawn and progressively norms were set to regulate accreditation of institutions and procedures [8]. The cancer care network in Brazil, in both the public and private sectors, from prevention to palliative care, includes primary care, home care, and specialised outpatient and hospital care, in addition to support systems, regulations, logistics, and governance [9]. The public Unified Health System (Sistema Único de Saúde–SUS) subsidises most cancer treatments because of their high cost. In addition, a part of the approximately 25% of the Brazilian population that has private health insurance [10] uses the SUS for cancer treatment [11].

Cancer treatment is performed in specialised care units, including (1) high-complexity oncology centres (Centros de Assistência de Alta Complexidade em Oncologia–CACONs), which treat all cancers, including haematological cancers, and may or may not treat paediatric cancers; (2) high-complexity oncology units (Unidades de Assistência de Alta Complexidade em Oncologia–UNACONs), which treat the most prevalent cancers with or without radiotherapy, haematology-oncology, and/or paediatric cancer services; and (3) hospital complexes (general hospitals with cancer surgery in the hospital complex, and radiotherapy services in the hospital complex), which perform specific complementary procedures and are affiliated with CACONs or UNACONs [12].

As determined in current regulations, these services should be distributed among the health administration regions (HARs) according to population criteria [12]. The HARs are groups of geographically continuous municipalities that share communication and transportation systems, present common characteristics, and establish partnerships to provide health actions and services [13].

The effects on the provision of oncology services of the historical structural rules and resources envisaged in Brazilian legislation [8] are not well known. The objective of this study was to analyse the structure of cancer care envisaged in Brazilian norms, describe the types of accreditation of cancer services and their geographic distribution, and determine the planning and evaluation parameters used to license healthcare facilities that provide cancer care in Brazil.

## Methods

This observational study identified the current norms for the organisation of cancer care and of all health services accredited by the SUS for cancer treatment in February 2017.

An initial analysis of the existing structural rules and resources was conducted. The analysis was grounded on Giddens’ structuration theory, which is based on the duality of structure, in which the structural properties of the social system are both the means and the result of social practices. Giddens considers that social practices are procedures executed from structural rules and resources [14]. The rules have normative aspects related to rights and obligations and semantic aspects related to the procedural and qualitative interpretation of care practices. Resources can be authoritative by the empowerment of subjects or institutions or allocative by the supply of materials [14]. Giddens’ theory has been previously used in analyses of the healthcare system in Brazil [15].

The institutional analysis proposed in this study emphasises the structural properties of the health system, considering the ways in which the structure, via rules (laws, norms, and protocols) and resources (human, financial, structural, and authoritative), facilitate or restrict [14] the organisation of cancer care in Brazil. In the moment of analysis had five norms in force [9, 12, 16–18] for cancer care according to the precepts of Giddens’ structuration theory.

To characterise the services, the following data were collected from the norms published by the Ministry of Health (MH) [19, 20] and the National Register of Health Establishments (Cadastro Nacional de Estabelecimentos de Saúde–CNES) [21]: geographical location (municipality and state), type of accreditation (CACON, UNACON, or hospital complex), type of service (clinical oncology, haematology-oncology, paediatric oncology, radiotherapy, and surgical oncology), and hospital classification according to the number of cancer surgeries performed annually (size A, > 999; size B, 600–999; and size C, < 600). The geographic macroregion (north, northeast, centre-west, south, and southeast) and HAR in which the health establishment was based were also identified.

Type of accreditation by MoH (as regulated by the Brazilian norm) is exclusively based on facility structure, types of services offered by facility, and human resources [12]. We considered all accredited facilities by SUS. No distinction between services, as to private or public nature was made.

The HARs were grouped as proposed by Viana et al. [22]: group 1, low socioeconomic status and low supply of health services; group 2, middle/high socioeconomic status and low supply of health services; group 3, middle socioeconomic status and intermediate supply of health services; group 4, high socioeconomic status and intermediate supply of health services; and group 5, high socioeconomic status and high supply of health services. The classification criteria of each macroregion were extracted from the website “Region and Networks - Path of universalization of health in Brazil” [23].

The adequacy between the number of licensed health services and healthcare needs was evaluated according to population size, as recommended by ordinance no. 140/2014 [12]. Based on the established norm, the planning and evaluation parameter used in accreditation as CACON or UNACON in Brazil was one accredited establishment per 500,000 inhabitants in each HAR. For the provision of haematology-oncology and paediatric oncology services, the norm recommended one establishment for every 3,500,000 inhabitants in the north; 2,700,000 inhabitants in the northeast; 1,700,000 inhabitants in the centre-west; and 1,300,000 inhabitants in the southeast and south macroregions [12]. Adequacy levels were classified as follows: adequate—number of licensed services is as required; higher—number of licensed services is greater than required; lower—number of licensed services is less than required. This analysis was separated by state because in 2017 the HARs did not cross state borders, and the evaluation using this approach was deemed pertinent. Population data were extracted from the 2017 population estimate of municipalities and states published by the Brazilian Institute of Geography and Statistics (Instituto Brasileiro de Geografia e Estatística–IBGE) [24].

The number of accredited health services in each HAR was measured according to the type of accreditation and type of health service to determine variations between different strata. For that purpose, three cutoff points were used on the basis of the number of accredited facilities: 1, 2–5, and > 5.

## Results

The analysis of the norms indicated that these documents served as structuring rules and resources for developing and implementing cancer care policies in Brazil.

The norms in force in 2017 for cancer care according to the precepts of Giddens’ structuration theory are summarised in Table 1.

**Table 1:** Institutional analysis of cancer care regulations in Brazil based on the principles of Giddens’ structuration theory

Structural property	Aspect	Regulation	Type	Identified characteristic
Rules	Normative	PNPCC	Facilitative	Establishes criteria for organizing the healthcare network
		Ordinance no. 2947/2012	Facilitative	Establishes criteria for the accreditation of hospitals for cancer surgeries
		Ordinance no. 140/2014	Facilitative	Establishes criteria for the accreditation of hospitals for high-complexity procedures in oncology
	Semantic	PNPCC	Restrictive	Establishes care of paediatric patients and patients with rare tumours in facilities qualified as CACON
Resources	Allocative	Ordinance no. 2947/2012	Facilitative	Financial increase for hospitals of size A and B
		Ordinance no. 931/2012	Facilitative	Installation of radiotherapy machines
		Ordinance no. 1357/2017	Restrictive	Withdraws investments for the creation and maintenance of strategic public health surveillance services
	Authoritative	Ordinance no. 2947/2012	Restrictive	Closing of hospitals of size C that do not increase the number of cancer surgeries
		Ordinance no. 140/2014	Restrictive	Differential planning and evaluation parameters for paediatric oncology and haematology-oncology services in the north, northeast, and centre-west regions of Brazil. Limits the number of accreditations for establishments with good infrastructure and that previously received financial and human resources.

*CACON: High-Complexity Oncology Centre; PNPCC: National Policy for Cancer Prevention and Control*

The documentary analysis identified 319 facilities, corresponding to 299 high-complexity oncology accreditations provided by the SUS. The discrepancy between the number of facilities and the number of licenses was due to the grouping of two or more facilities to form a single hospital complex or to additional clinical oncology services offered in an accredited facility, as provided for in the norms.

Most of the identified accreditations were the UNACON type (27.4%), followed by UNACON with radiotherapy services (20.1%). Facilities accredited solely for haematology-oncology and facilities accredited solely for paediatric oncology were identified (Table 2).

**Table 2:** Distribution of the types of accreditation of oncology services. Brazil, 2017

Type of accreditation	n (%)
CACON	18 (6.0)
CACON with paediatric oncology service	26 (8.7)
UNACON	82 (27.4)
UNACON with radiotherapy service	60 (20.1)
UNACON with haematology-oncology service	23 (7.7)
UNACON with paediatric oncology service	7 (2.3)
UNACON with radiotherapy and haematology-oncology services	33 (11.0)
UNACON with radiotherapy and paediatric oncology services	3 (1.0)
UNACON with paediatric oncology and haematology-oncology services	8 (2.7)
UNACON with radiotherapy, paediatric oncology and haematology-oncology services	13 (4.3)
UNACON exclusive for haematology-oncology services	2 (0.7)
UNACON exclusive for paediatric oncology services	13 (4.3)
UNACON exclusive of paediatric oncology with radiotherapy service	2 (0.7)
Radiotherapy services in the hospital complex	1 (0.3)
General hospital with cancer surgery in the hospital complex	8 (2.7)
Total	299 (100.0)

*CACON: High-Complexity Oncology Centre; UNACON: High-Complexity Oncology Unit*

The distribution by municipality indicated that the health facilities were located in 173 (3.1%) of the 5570 existing municipalities, and 39.4% were concentrated in state capitals. All Brazilian macroregions and states had at least one accredited facility. In some states, there were no facilities licensed in radiotherapy (Amapá and Roraima), paediatric oncology (Acre, Amapá, Maranhão, Rondônia, Roraima, and Tocantins), or haematology-oncology (Acre, Amapá, Rondônia, Roraima, and Tocantins) (Table 3).

**Table 3:** Distribution of the accreditation of oncology services and specific cancer service types by geographic location. Brazil, 2017

Geographic location	Accreditation of oncology services n (%)	Type of oncology service				
		Clinical oncology n (%)	Radiotherapy n (%)	Surgical oncology n (%)	Paediatric oncology n (%)	Haematology-oncology n (%)
North	**12 (4.0)**	**10 (3.7)**	**8 (5.1)**	**10 (3.7)**	**2 (2.8)**	**2 (1.6)**
Acre	1 (0.3)	1 (0.4)	1 (0.6)	1 (0.4)	–	–
Amazonas	3 (1.0)	1 (0.4)	2 (1.3)	2 (0.7)	1 (1.4)	1 (0.8)
Amapá	1 (0.3)	1 (0.4)	–	1 (0.4)	–	–
Pará	2 (0.7)	2 (0.7)	2 (1.3)	2 (0.7)	1 (1.4)	1 (0.8)
Rondônia	2 (0.7)	2 (0.7)	2 (1.3)	1 (0.4)	–	–
Roraima	1 (0.3)	1 (0.4)	–	1 (0.4)	–	–
Tocantins	2 (0.7)	2 (0.7)	1 (0.6)	2 (0.7)	–	–
Northeast	**57 (19.1)**	**52 (19.1)**	**27 (17.3)**	**50 (18.6)**	**14 (19.4)**	**20 (16.3)**
Alagoas	5 (1.7)	4 (1.5)	3 (1.9)	5 (1.9)	2 (2.8)	2 (1.6)
Bahia	14 (4.7)	13 (4.8)	8 (5.1)	13 (4.8)	2 (2.8)	4 (3.3)
Ceará	9 (3.0)	8 (2.9)	4 (2.6)	9 (3.3)	2 (2.8)	5 (4.1)
Maranhão	3 (1.0)	3 (1.1)	2 (1.3)	3 (1.1)	–	2 (1.6)
Paraíba	4 (1.3)	4 (1.5)	2 (1.3)	4 (1.5)	2 (2.8)	1 (0.8)
Pernambuco	10 (3.3)	9 (3.3)	3 (1.9)	8 (3.0)	2 (2.8)	3 (2.4)
Piauí	3 (1.0)	3 (1.1)	1 (0.6)	1 (0.4)	1 (1.4)	1 (0.8)
Rio Grande do Norte	7 (2.3)	6 (2.2)	2 (1.3)	5 (1.9)	2 (2.8)	1 (0.8)
Sergipe	2 (0.7)	2 (0.7)	2 (1.3)	2 (0.7)	1 (1.4)	1 (0.8)
Southeast	**141 (47.2)**	**126 (46.3)**	**75 (48.1)**	**127 (47.2)**	**36 (50.0)**	**63 (51.2)**
Espírito Santo	7 (2.3)	7 (2.6)	2 (1.3)	6 (2.2)	1 (1.4)	5 (4.1)
Minas Gerais	33 (11.0)	33 (12.1)	25 (16.0)	31 (11.5)	3 (4.2)	17 (13.8)
Rio de Janeiro	27 (9.0)	22 (8.1)	11 (7.1)	18 (6.7)	6 (8.3)	8 (6.5)
São Paulo	74 (24.7)	64 (23.5)	37 (23.7)	72 (26.8)	26 (36.1)	33 (26.8)
South	**67 (22.4)**	**64 (23.5)**	**35 (22.4)**	**62 (23.0)**	**15 (20.8)**	**32 (26.0)**
Paraná	24 (8.0)	23 (8.5)	11 (7.1)	22 (8.2)	6 (8.3)	11 (8.9)
Rio Grande do Sul	28 (9.4)	28 (10.3)	17 (10.9)	26 (9.7)	7 (9.7)	16 (13.0)
Santa Catarina	15 (5.0)	13 (4.8)	7 (4.5)	14 (5.2)	2 (2.8)	5 (4.1)
Centre-West	**22 (7.4)**	**20 (7.4)**	**11 (7.1)**	**20 (7.4)**	**5 (7.0)**	**6 (4.9)**
Federal District	5 (1.7)	4 (1.5)	2 (1.3)	4 (1.5)	1 (1.4)	1 (0.8)
Goiás	5 (1.7)	5 (1.8)	3 (1.9)	5 (1.9)	1 (1.4)	2 (1.6)
Mato Grosso	5 (1.7)	5 (1.8)	2 (1.3)	5 (1.9)	2 (2.8)	2 (1.6)
Mato Grosso do Sul	7 (2.3)	6 (2.2)	4 (2.6)	6 (2.2)	1 (1.4)	1 (0.8)
Brazil	**299 (100)**	**272 (100)**	**156 (100)**	**269 (100)**	**72 (100)**	**123 (100)**

With respect to the classification of authorised hospitals according to the number of annual cancer surgeries, 56 (18.7%) were size A, 56 (18.7%) were size B and 155 (51.8%) were size C. Two facilities (0.7%) were authorised to perform cancer surgeries but were not classified by the MH norms. Thirty facilities (10.1%) were not included in this classification because they did not perform cancer surgery.

**Fig. 1:**
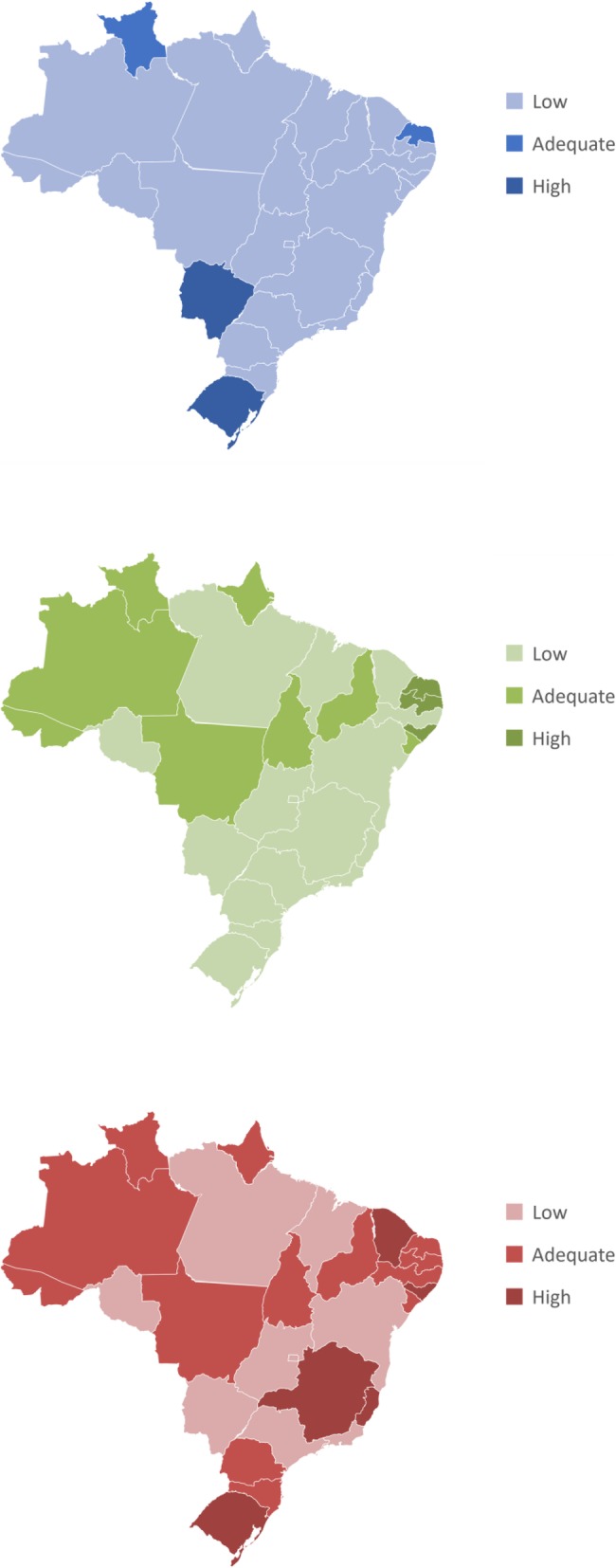
Level of adequacy of planning and evaluation parameters for oncology services by geographical location. Brazil, 2017. **a** Facilities licensed as CACON or UNACON. **b** Facilities with paediatric oncology services. **c** Facilities with haematology-oncology services. *CACON: High-Complexity Oncology Centre; UNACON: High-Complexity Oncology Unit. **Levels of adequacy: Adequate, number of licensed services as required; High, number of licensed services greater than required; Low, number of licensed services less than required. *** Maps drawn by authors

Considering the planning and evaluation parameters and an estimated Brazilian population of 207,660,929 inhabitants in 2017, there was a deficit of 144 CACON- or UNACONs-type services, 53 paediatric oncology services, and two haematology-oncology services. The relationship between supply and demand of accredited health services in Brazil is shown in Fig. 1 a, b, and c. The distribution of accredited cancer care facilities was heterogeneous and varied by the type of care, and care services were concentrated in a few states. The states in the south had the highest need versus supply ratio, and the states in the north had the lowest ratio.

The analysis of the HARs indicated that 153 (34.9%) of the 438 existing facilities had at least one accredited service. Proportionally, the number of facilities authorised to provide cancer services followed the distribution of the health regions. Based on population data, 124 regions (28.3%) had deficits in CACONs or UNACONs. Most facilities (56.9%) were located in HARs of group 5. The HARs of groups 1 and 2 had a relatively lower variability of specific types of services and did not have accredited paediatric oncology services (Table 4).

**Table 4:** Dimensional analysis of planning and evaluation parameters according to the health administration region type. Brazil, 2017

Dimensional analysis	Type of health administration region					
	Group 1	Group 2	Group 3	Group 4	Group 5	Total
	(*n* = 175)	(*n* = 47)	(*n* = 129)	(*n* = 27)	(*n* = 60)	(*n* = 438)
Number of geographical regions with facilities authorized for oncology services	14 (8.0%)	8 (17.0%)	58 (45.0%)	17 (62.9%)	55 (91.7%)	153 (34.9%)
Number of CACON^(d)^ or UNACON^(e)^ facilities according to planning and evaluation parameters						
*Adequate*	109	37	85	14	29	274
*Higher than necessary*	2	1	19	3	15	40
*Lower than necessary*	64	9	25	10	16	124
Number of paediatric oncology services^(f)^ according to planning and evaluation parameters						
*Adequate*	174	47	117	18	27	383
*Higher than necessary*	0	0	6	2	18	26
*Lower than necessary*	1	0	6	7	15	29
Number of haematology-oncology services^(g)^ according to planning and evaluation parameters						
*Adequate*	173	47	108	16	21	365
*Higher than necessary*	1	0	17	5	32	55
*Lower than necessary*	1	0	4	6	7	18
Number of facilities according to oncology service accreditation type						
*CACON*^*(a)*^	1	0	8	4	31	44
*UNACON*^*(b)*^	15	8	66	24	133	246
*Hospital complexes*^*(c)*^	0	0	0	3	6	9
Number of specific cancer services by type						
*Clinical oncology*	16	8	73	28	148	273
*Paediatric oncology*	0	0	8	7	57	72
*Haematology-oncology*	1	0	24	12	86	123
*Radiotherapy*	7	2	46	15	86	156
*Surgical oncology*	12	8	66	28	155	269
Number of facilities authorized as CACON^(a)^ or UNACON^(b)^						
*1*	14	8	48	13	21	104
*2 to 5*	1	0	11	5	28	45
*> 5*	0	0	0	0	6	6

*(a) CACON: High-Complexity Oncology Centre, including the subcategory CACON with paediatric oncology; (b) UNACON: High-Complexity Oncology Unit, including the subcategories UNACON with paediatric oncology, UNACON with haematology-oncology, UNACON with radiotherapy, UNACON exclusive for paediatric oncology, and UNACON exclusive for haematology-oncology; (c) Includes radiotherapy service in the hospital complex and general hospital with cancer surgery in the hospital complex; (d) Includes only the high-complexity oncology centre; (e) High-complexity oncology unit, including the subcategories UNACON with paediatric oncology, UNACON with haematology-oncology, and UNACON with radiotherapy; (f) Includes CACON with paediatric oncology, UNACON with paediatric oncology, and UNACON exclusive for paediatric oncology; (g) Includes CACON, CACON with paediatric oncology, UNACON with haematology-oncology, and UNACON exclusive for haematology-oncology*

## Discussion

The results allow a detailed discussion about the analytical categories contemplated in the study objectives. These categories may provide an overview of the structure of cancer care in Brazil on the basis of the principles of Giddens’ structuration theory [14]. As the Brazilian experience is used in international exchanges with low- and middle-income developing countries, this analysis may be useful to these countries when organising their cancer care networks [25].

The results indicated that the institutionalisation of healthcare policies was sustained by stimulating the expansion of care services and accreditation by sanctioning norms [14]. This approach has historically been used in Brazil, when the structuring rules of cancer care were established after the creation of the SUS [8].

Notably, circumstances may transform a facilitative rule into a restrictive rule. Ordinance no. 140/2014 [12], in defining the requirements for the functioning of specialised care services and establishing organisational and management criteria and parameters, has proved to be an authoritative resource according to the precepts of Giddens’ structuration theory [14]. Therefore, no allocative resources were established to ensure compliance with the established rules, which thus prevented compliance by many institutions. Although the norm can be considered a facilitator, establishing a set of criteria that qualify services may be restrictive by accrediting only establishments that already have the infrastructure and financial and human resources necessary to meet the established requirements. This bias directly affects the expansion of the healthcare network, with a higher impact on CACON-type licenses because of the higher level of demand. A similar situation was observed with ordinance no. 2947/2012 [17] and with the National Policy for Cancer Prevention and Control (PNPCC) [9].

Ordinance no. 2947/2012 [17] is considered a normative rule because it defines the relationship between the number of performed cancer surgeries and hospital size (A, B, or C). For hospitals of size A and B, this ordinance is also an allocative resource because it establishes a 20% increase in the remuneration for hospital services and professional services related to performed surgeries, provided that an annual increase in productivity is demonstrated. Therefore, for these two categories, this norm has a facilitating effect [14] for the expansion of health services.

For hospitals of size C, the same norm [17] is restrictive [14] because it establishes the need to increase annual production by 25% and perform at least 600 surgical procedures annually for up to five years without any financial increase under the risk of disqualification at the end of this period. According to data from the analysed norm [19], approximately 52% of the hospitals were classified as size C. More than half of these establishments performed fewer than 400 cancer surgeries per year. For the two unclassified hospitals, it was not possible to identify the number of performed procedures to justify their classification. Changes to this norm may be necessary to improve compliance with the established parameters and avoid disqualifying these establishments and prevent interruptions in care services.

With respect to the type of accreditation of cancer services, most services were of the UNACON type. CACONs function as regional reference centres for treating all cancers [9], and our analysis indicated that there were only 44 licensed CACONs in Brazil, of which 70.5% were located in the south and southeast regions. A previous study found that the number of these type of accreditation has not increased over time [8]. Only CACONs are obliged to function as training centres for physicians and other health professionals in cancer care [12]. One shortcoming stemming from the small number of CACONs is the limitation in training, which probably has considerable impact on services.

One issue that needs to be addressed is that according to the PNPCC [9], CACONs are not obliged to treat rare and childhood cancers. Therefore, organisational models with an adequate flow of care for patients with rare tumours involving the whole cancer care network need to be developed to guarantee the timely execution of the most appropriate treatment possible [26]. The same approach should be used in paediatric patients aged 0–19 years [7]. Although there are reference centres for treating adolescent patients, this population is outside the age groups most commonly affected by cancer and is not assisted by professionals of many specialties [27].

According to the principles of Giddens’ structuration theory [14], the procedural and qualitative semantic aspects of care practices expressed in the PNPCC [9] indicate a commitment to the care of paediatric patients and patients with rare tumours; however, the care lines for treating these cancers are not fully defined in Brazil, and the therapeutic itinerary these patients must follow is unclear. Additionally, from a semantic point of view, primary health care is only responsible for screening and detection of signs and symptoms of cancer, and is not involved in diagnosis, or follow-up or treatment. Diagnosis and treatment are done in secondary and tertiary facilities. Regional hospitals may eventually give support to patients - they may manage symptoms - but only if SUS-accredited are part of the cancer care network and directly treat cancer.

Another aspect related to service accreditation is the multimodality of cancer treatment, which should be performed simultaneously or consecutively regardless of whether it is curative or palliative [28] and requires multiprofessional and intersectional actions to ensure that all treatments are performed as planned. Most establishments were licensed as UNACONs, resulting in the need to refer patients to external radiotherapy services, which may affect the optimal treatment time and compromise the therapy effectiveness. However, the status of cancer care in Brazil is poorly understood because of the limited availability of national data on cancer treatment outcomes and their correlation with institutional investments over time [29].

With regard to the geographic distribution of oncology services, the results revealed that the number of services authorised for cancer care in Brazil is small. The most critical region is the north, with only 12 accredited facilities, including 11 high-complexity cancer services.

The number of authorised hospitals in state capitals (approximately 40.0%) should be considered, even though it remains insufficient coverage because of the large size of the local population. The distance travelled by patients residing in rural areas given the territorial dimensions of Brazil is a significant challenge and may compromise the access, continuity and comprehensiveness of care. Problems related to the flow of services for adults and children with cancer have been evidenced in previous studies [30, 31], which stressed the long distances travelled by patients to hospitals providing oncology services.

Although the distance travelled by patients is one of the many problems affecting patient access to oncological services, addressing this problem is essential to improve health planning and ensure an adequate allocation of financial resources. In the United States, approximately 50% of the population is located less than one hour from a referral service for cancer treatment [32], which improves treatment outcomes in the country.

Another aspect to be addressed is the limited access to cancer services, especially to radiotherapy, haematology-oncology, and paediatric oncology. The high variability in the distribution of these services indicates that the implementation of new services is not considering the regions with the highest deficits in specialised care.

Radiotherapy is critical because approximately 50% of cancer patients undergo this type of therapy at some stage of treatment [33]. Problems in the coverage of radiotherapy services have been reported in several countries, with an estimated deficit of more than 7000 radiotherapy machines, resulting in approximately 2 million people without treatment, especially in low- and middle income developing countries [34], including Brazil. The “Plan for the Expansion of Radiation Therapy in the SUS” was established in 2012 [16] to minimise this deficit in Brazil by creating or expanding health services. According to the norm, the MH was responsible for financing the purchase and installation of 80 new machines; therefore, this norm was characterised as a facilitating allocative resource [14]. However, Araújo et al. [35] found that more than 250 radiotherapy services were necessary by 2015, and projections for 2030 indicated a shortage of approximately 200 new radiotherapy machines in Brazil under the current financial investments. In addition, the distribution of machines should be evaluated to increase the access of the population to radiotherapy. Santibáñez et al. [36] reported that the maximum distance travelled to reach radiotherapy services should be no more than 90 min and that times longer than this decreased the use of these services, with significant clinical consequences.

With regard to paediatric oncology, treatment abandonment due to socioeconomic problems, insufficient provision of health services, and worsening of patient quality of life are the leading causes of therapeutic failure worldwide [37]. In Brazil, it is estimated that most paediatric oncology services are provided by facilities that are not accredited for this purpose, which may represent a higher risk for the individuals undergoing cancer treatment [31, 38]. Specific norms for paediatric services are absent, and more investments are necessary to improve the care network for paediatric cancer diagnosis and care.

The treatment of haematologic malignancies requires specific treatment centres because of the complexity and specificity of the tumours [39]. Ensuring comprehensive treatment of the affected patients is critical because these tumours are highly curable [40]. Although the number of haematology-oncology services in Brazil is close to that recommended by the norms, little is known about the quality, the complexity of the services offered, and the treatment outcomes.

It is critical to question the motivation of using different population parameters for each Brazilian macroregion to assess the need for haematology-oncology and paediatric oncology services. This authoritative resource [14] seems to play a restrictive role in the north, northeast, and centre-west, promoting greater inequity in these regions. The deficits identified would be higher if the same population parameters were used in all macroregions. For instance, in Acre, there are no accredited haematology-oncology or paediatric oncology services; however, when the calculation parameters were applied, the number of accredited services was considered adequate (Fig. 1 b and c). This incoherence demonstrates the limitations of the suggested approach.

Although most facilities performed cancer surgery, it is essential to reflect on the adequacy of services and the rules established by the current norms. It is estimated that more than 80% of cancer patients will require surgery during treatment [41]. However, it is necessary to adopt strategies that favour patient access to surgical procedures, improve professional training, increase the safety of these procedures, and integrate surgical centres into facilities that perform other treatment modalities [2, 41]. This integration needs to be done in Brazil because few facilities are qualified as general hospitals with cancer surgery in the hospital complex.

The planning of cancer care should consider the minimum recommended parameters to establish higher equity and promote the comprehensiveness of care [4, 5]. However, the parameters currently used to assess the need for accredited services in Brazil should be questioned because the best basis of calculation of the number of services is cancer incidence or disease burden, not population size [42, 43]. Nevertheless, the use of incidence data may mask the actual need for these services because these data are produced based on diagnosis, in which secondary care is effective, and this is why it is estimated that population parameters are a good option in the north and northeast macroregions. Furthermore, the use of incidence data may improve the distribution of cancer treatment centres in Brazil.

However, incidence data are not available for all municipalities and HARs in Brazil [7]. It is necessary to improve the cancer registry model to produce more reliable data in each HAR, allowing changes in the current parameters and enabling better planning of the SUS cancer care network. Registries have been useful in elucidating exposure differences throughout the country, which is supported by evidence [44]. Nonetheless, restrictive measures were recently identified with the withdrawal of investments for the creation and maintenance of strategic public health surveillance services [18], compromising the execution of cancer registration nationwide.

Another aspect to consider when establishing need is cancer screening. The Brazilian regulatory norm does not consider screening as a determining factor; in fact, does not even make mention of it [12]. However, evidence shows that although screening for breast and cervical cancer is present in the SUS, its availability has no bearing on distribution of cancer care services [45, 46].

Lastly, the analysis according to the type of HAR confirmed that high-complexity oncology services are more common in more developed, high-income regions. The provision of services in reference cities is feasible considering the economy of scale and scope predicted in the logic of the organisation of integrated healthcare systems [47]. However, this arrangement should consider the geographical accessibility of users, the epidemiological issues, the number of qualified professionals, and the availability and capacity of each facility [30, 36, 48] to improve access to services, especially by socioeconomically marginalised populations.

The deficit of accredited facilities in approximately 28% of the HARs is a cause for concern. The formation of health macroregions capable of meeting the care demands of two or more HARs seems to be necessary in this care model to increase the quality of care to cancer patients [49]. An adequate analysis of actual service needs could be undertaken if health macroregions were considered in the planning and evaluation parameters. However, data on Brazilian health macroregions are limited.

There is a large amount of evidence that cancer care outcomes can be affected by factors other than physical structure. However, our approach to systematising the conceptual elements of the structuration theory considered that SUS practices are regulated by several norms and regulations that are contextualised by actions and, therefore, affect these outcomes [50].

This study has some limitations. First, the analysis considered the implementation of normative policies rather than strategic behaviour analysis, which would have allowed evaluating the performance of each agent [15, 50]. Second, identifying health macroregions in the consulted data sources was not possible, which may limit the analysis of the planning and evaluation parameters. Third, the consulted norms only allowed identifying accredited establishments, but not health units undergoing accreditation that already provided cancer care. Fourth, quantifying licensed facilities may be insufficient to ensure adequate treatment to all healthcare users, as service capacity in different institutions varies according to the number of professionals, amount of equipment, and the number of patients under treatment and follow-up [51]. These variables strongly affect the ability to perform new procedures. Notwithstanding, it should be emphasised that the study attempted to evaluate the association between the number of establishments and population parameters as recommended by the norm in force.

These last three limitations suggest the natural bounds of the study due to the study design and the data sources used, but they are also indicative of a strength of the study. The established norms have not been effective for the adequate structuring of cancer care services in Brazil and consequently need to be revised, not just re-edited, as has been happening in Brazil in the past few years.

## Conclusion

There are major disparities in the availability of high-complexity oncology services and a high variability in the types of specific services available in Brazil. According to Giddens’ structuration theory, these aspects exemplify the control dialectic involving asymmetric access to means (resources), typical of social contexts. The identified deficits may strongly affect patient survival, quality of life, and cancer-related mortality.

Changes in the planning and evaluation parameters are critical to guarantee universality, equity in access to healthcare, and comprehensiveness of care for cancer patients. The norms and resources used in the formulation of policies should be more inductive and facilitate improvements in cancer care availability and quality in Brazil.

## Data Availability

Data used in this analysis is available online from Brazilian National Register of Health Establishments - http://cnes.datasus.gov.br/pages/consultas.jsp. This is a public free-access databank of the Brazilian Ministry of Health.

## Abbreviations

CACON: High-complexity oncology centres (Centros de Assistência de Alta Complexidade em Oncologia)
CNES: National Register of Health Establishments (Cadastro Nacional de Estabelecimentos de Saúde)
HAR: Health administration regions
IARC: International Agency for Research on Cancer
IBGE: Brazilian Institute of Geography and Statistics (Instituto Brasileiro de Geografia e Estatística)
MH: Ministry of Health
PNPCC: National Policy for Cancer Prevention and Control
SUS: Unified Health System (Sistema Único de Saúde)
UNACON: High-complexity oncology units (Unidades de Assistência de Alta Complexidade em Oncologia)

## References

[1] Bray F, Ferlay J, Soerjomataram I, Siegel RL, Torre LA, Jemal A (2018). Global cancer statistics, 2018: GLOBOCAN estimates of incidence and mortality worldwide for 36 cancers in 185 countries. CA Cancer J Clin.

[2] Hoekstra HJ, Wobbes T, Heineman E, Haryono S, Aryandono T, Balch CM (2016). Fighting global disparities in cancer care: a surgical oncology view. Ann Surg Oncol.

[3] Souza JA, Hunt B, Asirwa FC, Adebamowo C, Lopes G (2015). Global health equity: cancer care outcome disparities in high-, middle-, and low-income countries. J Clin Oncol.

[4] Karanikolos M, Ellis L, Coleman MP, McKee M (2013). Health systems performance and cancer outcomes. J Natl Cancer Inst Monogr.

[5] Coleman MP (2014). Cancer survival: global surveillance will stimulate health policy and improve equity. Lancet.

[6] Fitzmaurice C, Allen C, Barber RM, Barregard L, Bhutta ZA, Brenner H (2017). Global, regional, and national cancer incidence, mortality, years of life lost, years lived with disability, and disability-adjusted life-years for 32 cancer groups, 1990 to 2015: a systematic analysis for the global burden of disease study. JAMA Oncol.

[7] Brazil. National Cancer Institute (Instituto Nacional de Câncer -INCA). Estimate 2018: cancer incidence in Brazil (Estimativa 2018: incidência de câncer no Brasil). Rio de Janeiro: INCA; 2017.

[8] Silva MJS, Lima FLT, O’dwyer G, Osorio-de-Castro CGS (2017). Política de Atenção ao Câncer no Brasil após a criação do Sistema Único de Saúde. Rev Bras Cancerol.

[9] Brazil. Ministry of Health. Consolidation Ordinance no. 2 (Attachment IX): Provides for the National Policy for Cancer Prevention and Control (Dispõe sobre a Política Nacional para Prevenção e Controle do Câncer).: Official Press (Diário Oficial da União); 2017.

[10] Strasser-Weippl K, Chavarri-Guerra Y, Villarreal-Garza C, Bychkovsky BL, Debiasi M, Liedke PE (2015). Progress and remaining challenges for cancer control in Latin America and the Caribbean. Lancet Oncol.

[11] Brazil. National Regulatory Agency for Private Health Insurance and Plans (Agência Nacional de Saúde Suplementar -ANS). Newsletter: use of the public system by beneficiaries of health plans and reimbursement to SUS (Boletim informativo: utilização do sistema público por beneficiários de planos de saúde e ressarcimento ao SUS). Rio de Janeiro: ANS; 2017.

[12] Brazil. Ministry of Health. Ordinance no. 140: Redefines the criteria and parameters for organization, planning, monitoring, control and evaluation of accreditated health care establishments in specialized oncology care and defines the structural, operational and human resources conditions for the accreditation of these establishments within the Unified Health System (SUS) (Redefine os critérios e parâmetros para organização, planejamento, monitoramento, controle e avaliação dos estabelecimentos de saúde habilitados na atenção especializada em oncologia e define as condições estruturais, de funcionamento e de recursos humanos para a habilitação destes estabelecimentos no âmbito do Sistema Único de Saúde (SUS)).: Official Press (Diário Oficial da União); 2014.

[13] Brazil. Presidency of the Republic. Decree no. 7,508: Regulates Law no. 8,080, dated 19 Sep 1990, to provide for the organization of the Unified Health System - SUS, health planning, health care and interfederative articulation, and other measures (Regulamenta a Lei n.° 8.080, de 19 de setembro de 1990, para dispor sobre a organização do Sistema Único de Saúde – SUS, o planejamento da saúde, a assistência à saúde e a articulação interfederativa, e dá outras providências).: Official Press (Diário Oficial da União); 2011.

[14] Giddens A (2003). A constituição da sociedade.

[15] O'dwyer G, Konder MT, Machado CV, Alves CP, Alves RP (2013). The current scenario of emergency care policies in Brazil. BMC Health Serv Res.

[16] Brazil. Ministry of Health. Ordinance no. 931: Establishes the Plan for the Expansion of Radiation Therapy in the Unified Health System (SUS) (Institui o Plano de Expansão da Radioterapia no Sistema Único de Saúde (SUS)).: Official Press (Diário Oficial da União); 2012.

[17] Brazil. Ministry of Health. Ordinance no. 2,947: Updates, by exclusion, inclusion and alteration, oncological surgical procedures in the Table of Procedures, Medications, Orthoses / Prostheses and Special Materials of SUS (Atualiza, por exclusão, inclusão e alteração, procedimentos cirúrgicos oncológicos na Tabela de Procedimentos, Medicamentos, Órteses/Próteses e Materiais Especiais do SUS).: Official Press (Diário Oficial da União); 2012.

[18] Brazil. Ministry of Health. Ordinance no. 1,357: Disaccreditation federated entities to receive the costing financial incentive for implementation and maintenance of actions and strategic public services of health surveillance (Desabilita os entes federados ao recebimento do incentivo financeiro de custeio para implantação e manutenção de ações e serviços públicos estratégicos de vigilância em saúde).: Official Press (Diário Oficial da União); 2017.

[19] Brazil. Ministry of Health. Ordinance no. 3,398: Publishes the list of accredited hospitals in the High-Complexity Oncology classified as size A, B or C (Publica a relação de hospitais habilitados na Alta Complexidade em Oncologia classificados nos portes A, B ou C).: Official Press (Diário Oficial da União); 2016.

[20] Brazil. Ministry of Health. Ordinance no. 458: Maintain the accreditations of health establishments in High Complexity (Mantem as habilitações de estabelecimentos de saúde na Alta Complexidade).: (Diário Oficial da União); 2017.

[21] Brazil. National Register of Health Establishments (Cadastro Nacional dos Estabelecimentos de Saúde). http://cnes.datasus.gov.br/pages/consultas.jsp. Accessed 05 Jul 2017.

[22] Viana ALD, Bousquat A, Pereira APCDM, Uchimura LYT, Albuquerque MVD, Mota PHDS (2015). Typology of health regions: structural determinants of regionalization in Brazil. Saude Soc.

[23] Brazil. Region and Networks - Path of universalization of health in Brazil (Região e Redes – Caminho da universalização da saúde no Brasil). http://www.resbr.net.br/. Accessed 10 Aug 2017.

[24] Brazil. Brazilian Institute of Geography and Statistics (Instituto Brasileiro de Geografia e Estatística – IBGE). https://ww2.ibge.gov.br/home/estatistica/populacao/estimativa2017/estimativa_dou.shtm. Accessed 20 Aug 2017.

[25] Sirohi B, Chalkidou K, Pramesh CS, Anderson BO, Loeher P, El Dewachi O (2018). Developing institutions for cancer care in low-income and middle-income countries: from cancer units to comprehensive cancer centres. Lancet Oncol..

[26] Ray-Coquard I, Lauraine EP, Le Cesne A, Pautier P, Lavenue MCV, Trama A (2017). Improving treatment results with reference centres for rare cancers: where do we stand?. Eur J Cancer.

[27] Alvarez E, Keegan T, Johnston EE, Haile R, Sanders L, Saynina O (2017). Adolescent and young adult oncology patients: disparities in access to specialized cancer centers. Cancer..

[28] Onukwugha E, Petrelli NJ, Castro KM, Gardner JF, Jayasekera J, Goloubeva O (2016). Impact of multidisciplinary care on processes of cancer care: a multi-institutional study. J Oncol Pract..

[29] Siqueira ASE, Gonçalves JG, Mendonça PEX, Merhy EE, Land MGP (2017). Economic impact analysis of Cancer in the health system of Brazil: model based in public database. Health Sci J.

[30] Oliveira EXG, Melo ECP, Pinheiro RS, Noronha CP, Carvalho MS (2011). Acesso à assistência oncológica: mapeamento dos fluxos origem-destino das internações e dos atendimentos ambulatoriais. O caso do cancer de mama. Cad Saúde Pública.

[31] Grabois MF, Oliveira EXG, Carvalho MS (2013). Assistência ao câncer entre crianças e adolescentes: mapeamento dos fluxos origem-destino no Brasil. Rev Saúde Pública.

[32] Onega T, Alford-Teaster J, Wang F (2017). Population-based geographic access to parent and satellite National Cancer Institute Cancer center facilities. Cancer..

[33] Zubizarreta EVDJ, Van Dyk J, Lievens Y (2017). Analysis of global radiotherapy needs and costs by geographic region and income level. Clin Oncol.

[34] Yap ML, Zubizarreta E, Bray F, Ferlay J, Barton M (2016). Global access to radiotherapy services: have we made progress during the past decade?. J Glob Oncol.

[35] Araújo LP, Sá NM, Atty ATM (2016). Necessidades Atuais de Radioterapia no SUS e Estimativas para o Ano de 2030. Rev Bras Cancerol..

[36] Santibáñez P, Gaudet M, French J, Liu E, Tyldesley S (2015). Optimal location of radiation therapy centers with respect to geographic access. Int J Radiation Oncol Biol Phys.

[37] Goh XTW, Tan YB, Thirumoorthy T, Kwan YH (2017). A systematic review of factors that influence treatment adherence in paediatric oncology patients. J Clin Pharm Ther.

[38] Magalhães IQ, Gadelha MIP, Macedo CD, Cardoso TC (2016). A Oncologia Pediátrica no Brasil: Por que há Poucos Avanços?. Rev Bras Cancerol..

[39] Matthey F, Parker A, Rule SAJ, Wimperis JZ, Ardeshna KM, Bird JM (2010). Facilities for the treatment of adults with haematological malignancies – 'Levels of Care': BCSH Haemato-oncology task force 2009. Hematology..

[40] Snowden JA, O'connell S, Hawkins J, Dalley C, Jack A, Mannari D (2017). Haematological cancers: improving outcomes. A summary of updated NICE service guidance in relation to specialist integrated Haematological malignancy diagnostic services (SIHMDS). J Clin Pathol.

[41] Sullivan R, Alatise OI, Anderson BO, Audisio R, Autier P, Aggarwal A (2015). Global cancer surgery: delivering safe, affordable, and timely cancer surgery. Lancet Oncol..

[42] Gomes Junior SCS, Almeida RT (2009). Modelo de simulação para estimar a infraestrutura necessária à assistência oncológica no sistema público de saúde. Rev Panam Salud Pública.

[43] Tai CG, Hiatt RA (2017). The population burden of cancer: research driven by the catchment area of a cancer center. Epidemiol Rev.

[44] Tucker TC (2019). Durbin EB.

[45] Nogueira MC, Fayer VA, Corrêa CSL, Guerra MR, Stavola BD, dos-Santos-Silva I, Bustamante-Teixeira MT. Inequities in access to mammographic screening in Brazil. Cad Saude Publica. 2019;35(6): e00099817. 10.1590/0102-311X00099817 31291424

[46] Costa RFA, Longatto-Filho A, Vazquez FL, Pinheiro C, Zeferino LC, Fregnani JHTG (2018). Trend analysis of the quality indicators for the Brazilian cervical cancer screening programme by region and state from 2006 to 2013. BMC Cancer.

[47] Chalkidou K, Marquez P, Dhillon PK, Teerawattananon Y, Anothaisintawee T, Gadelha CAG (2014). Evidence-informed frameworks for cost-effective cancer care and prevention in low, middle, and high-income countries. Lancet Oncol..

[48] Murage P, Crawford SM, Bachmann M, Jones A (2016). Geographical disparities in access to cancer management and treatment services in England. Health Place.

[49] Simeone WJ, Bingham J, Burke TW, Pisters PW (2013). Quality assessment across a national cancer network. J Oncol Pract.

[50] O’dwyer G, Baptista TWF, Azevedo CS, Machado CV (2015). Estudos de políticas e a teoria da estruturação de Giddens. Políticas, planejamento e gestão em saúde: abordagens e métodos de pesquisa.

[51] Ma X, Sauré A, Puterman ML, Taylor M, Tyldesley S (2016). Capacity planning and appointment scheduling for new patient oncology consults. Health Care Manag Sci.

